# Reinfection and re-revision rates of 113 two-stage revisions in infected TKA

**DOI:** 10.7150/jbji.43705

**Published:** 2020-04-27

**Authors:** Joris Bongers, Anouk M.E. Jacobs, Katrijn Smulders, Gijs G. van Hellemondt, Jon H.M. Goosen

**Affiliations:** 1 Department of Orthopaedic Surgery, Prosthetic Joint Infection Unit, Sint Maartenskliniek, Nijmegen, the Netherlands; 2 Sint Maartenskliniek Research, Sint Maartenskliniek, Nijmegen, the Netherlands

## Abstract

**Introduction**: Two-stage revision is the most frequently performed revision procedure of a (suspected) periprosthetic joint infection (PJI) after total knee arthroplasty (TKA). The reported results of this treatment show large variability between studies, ranging between 0 - 40 percent failure. The purposes of this study were to determine long term (1) reinfection rate, (2) re-revision rates for any reason, and (3) the reinfection rate of patients with positive cultures at reimplantation.

**Methods**: We prospectively followed and retrospectively reviewed 113 consecutive two-stage revision TKAs, performed between 2003 and 2013 in our clinic with a minimum follow-up of 2 years. Diagnosis of PJI was based on the major Musculoskeletal Infection Society criteria for PJI.

**Results**: After a mean follow-up of 94 months (range 24-172 months), infection recurred in 23 cases (23%). Of these, nine cases (9%) were defined as relapse (same micro-organism as index revision) and in 14 cases another causative was found (14%). In 11 patients debridement, antibiotics and retention of the prosthesis successfully eradicated the reinfection. After overall follow-up 17 patients (17%) underwent re-revision surgery, 11 patients (11%) due to an infection and 6 patients (6%) for aseptic reasons.

**Conclusions**: Treatment of a (suspected) infection of a TKA by a two-stage revision had acceptable results based on re-revision and re-infection rates in the long term (>5 years), resembling the short-term results (<2 years). Focussing on the cultures at the index two-stage revision, episodes of relapse and new infections during follow-up were almost equally divided. Reinfection rates were higher in cases with positive cultures at reimplantation. Patients should be counselled appropriately in this particular situation.

## Introduction

Prosthetic joint infection (PJI) is a feared complication with an incidence of up to 2% following primary total knee arthroplasty (TKA) 1,2. The absolute incidence of PJI is expected to rise with increasing numbers of primary and revision TKA 3. Treatment of PJI is challenging due to the complex pathogenesis with biofilm formation 4. Eradication of infection can only be accomplished with a combination of an appropriate surgical treatment and long-term antimicrobial treatment 5. Suppressive antibiotic treatment alone is reserved for patients who are unable or unwilling to undergo surgery 2. Regarding complete revision, two-stage revision is still the gold standard procedure for treating chronic PJI, recommended by the Infectious Diseases Society of America (IDSA) 6.

The reported results of this treatment show large variability between studies, ranging between 0 - 40 percent failure, with varying follow-up time and failure definition 7,8. Few studies have reported primarily on the long-term rate of septic and mechanical failure following a two-stage revision for the treatment of PJI following TKA 9,10. Petis et al. reported reinfection rates of 17% at long-term, while re-revision rates for all causes were 16% in the study by Haleem et al 9,10. These studies need verification and have not evaluated the reinfection rates of patients with positive cultures at reimplantation.

The aim of our study was to investigate long term (1) reinfection rates, (2) re-revision rates for any reason and (3) the reinfection rate of patients with positive cultures at the time of reimplantation.

## Methods

### Patient selection

In our institutional registry every revision TKA due to infection is followed-up prospectively. A retrospective review of this registry identified patients treated with two-stage revision for suspected PJI from June 2003 to September 2013. Eligible for inclusion were patients with a minimum follow-up of 24 months. The Sint Maartenskliniek Nijmegen investigational review board approved the first study proposal, prior to initiation.

**Figure 1:**
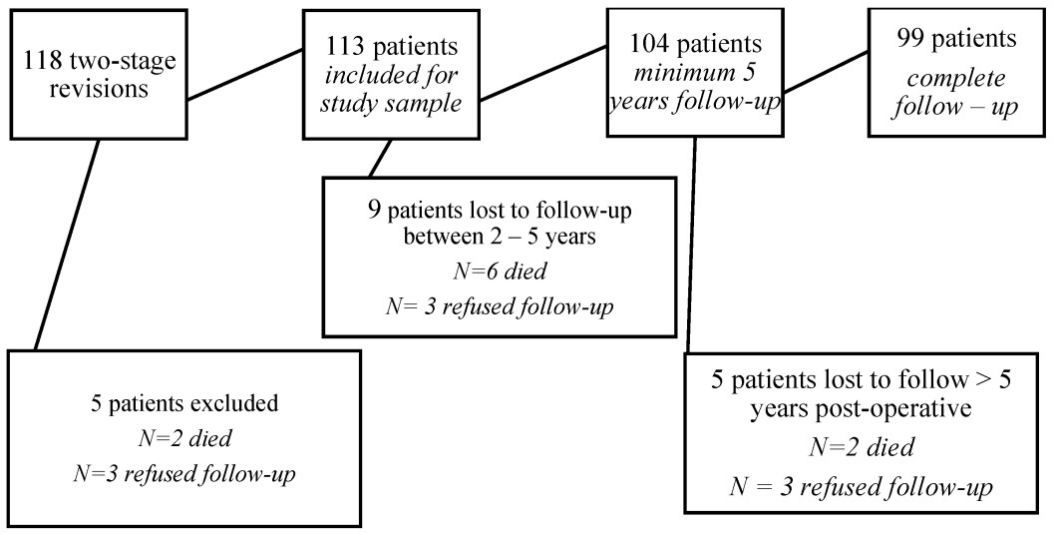
Flow diagram for patient inclusion and exclusion and patients lost to follow-up.

We identified 118 two-stage revisions for suspected PJI, performed by 7 experienced surgeons. Inclusion criteria were met by 113 patients. Patients were excluded if they died or refused follow-up before a minimum follow-up of 2 years. See Figure 1 for patient inclusion and exclusion and patients lost to follow-up.

### Study population

Of the 113 patients, 62 patients were female and the mean age at implant removal was 67.1 years (standard deviation (SD) 8.2). See Table 1 for patient characteristics, mean body mass index (BMI), and American Society of Anaesthesiologists (ASA) scores. In 34 (30%) of the cases a previous revision surgery was performed. Twenty patients (18%) underwent a previous two-stage revision for PJI. The mean follow-up was 7.8 years (range 2 - 14.3) following reimplantation.

### Diagnosis of infection

Sixty-six patients (58%) met at least 1 of the major MSIS (Musculoskeletal Infection Society) criteria for PJI 11. Because not all minor criteria were registered preoperatively in the time period of our data set, diagnosis of PJI was solely based on the major criteria: 2 positive cultures or the presence of a sinus tract. Patients undergoing two-stage revision who did not meet MSIS criteria, had a high clinical suspicion of PJI.

Sixty patients had 2 or more positive cultures with the same microorganism at implant removal (see Table 2). A polymicrobial infection was seen in 5 patients, all with 2 different microorganisms. In 21 patients a microorganism (35%) with a multidrug-resistant bacteria was cultured.

### Procedure

First-stage treatment consisted of implant and cement removal, obtaining a minimum of 6 tissue cultures for microbiology, extensive debridement, lavage (using 3 liters (L) of Betadine diluted Saline followed by 3L of Saline) and insertion of an antibiotic, non-articulating cement spacer. At first stage handmade mobile cementspacer of polymethyl methacrylate (PMMA) cement with 2gr gentamicin and/or 3 to 4 gr clindamycine/vancomycin per 40gr, based on preoperative culture results. Postoperatively, a knee immobilizer was applied for the duration of 1 week and continued afterwards with a cast until reimplantation.

In 6 patients, no cement spacer was implanted and an external fixator was placed due to soft-tissue compromise or severe bone loss. Two patients with an external fixator required reoperation before removal of the external fixator due to signs of ongoing infection. 2 patients with an external fixator had negative cultures at reimplantation; 4 patients with an external fixator had 1 positive culture at reimplantation. Fourteen 14 patients with a cement spacer underwent reoperation between first and second stage. In 10 patients exchange of spacer was required due to signs of ongoing infection. The other reasons for reoperation were wound necrosis and a dislocated spacer.

All patients received antibiotic treatment after intraoperative cultures were taken. Patients received cefazoline 3 grams per day until adjustment, based on the intraoperative cultures results, was indicated. In case of a cephalosporin allergy or cephalosporin-resistant organism, patients received vancomycin adjusted for BMI and kidney function or antibiotic treatment was based on previous cultivated susceptibility. To determine the antibiotic treatment, orthopaedic infectious disease specialists and microbiologists were consulted and recommended guidelines by the IDSA were followed 6. After cessation of antibiotic treatment for at least 2 weeks, patients underwent reimplantation. During reimplantation PMMA cement with 1 gr gentamicin and 1gr clindamycin (COPAL G+V, Heraeaus, Germany) or 0.5 gr gentamicin and 2 gr vancomycin (COPAL G+V, Heraeus, Germany) per 40g was used. Mean time between the 2 stages of surgery was 3.2 months (SD 1.9). After reimplantation, patients were treated with Cefazoline 3 grams per day. In case of 2 or more positive cultures after reimplantation, adjusted antibiotic therapy was continued for 3 months. In case of negative cultures or less than 2 positive cultures with the same organism, antibiotic therapy was continued for 6 weeks, based on the microorganism found at implant removal and guidelines described by Zimmerli et al 12. Patient follow-up included outpatient clinic visits at 2 and 6 weeks, 3 months, 1 year, 2 years, 5 years, and every 5 years thereafter.

### Microbiological method

In each patient a minimum of 6 specimens of periprosthetic tissue were taken before antibiotic treatment at time of implant removal and at reimplantation. Sonication of the removed implants was performed in only 33 patients (starting in 2011) and is no longer routine practice in our institution, therefore sonication results were not included in the study. Specimens were taken from femur and tibia, including the intramedullary canal, and were cultured for aerobic and anaerobic organisms with a minimum incubation time of 2 weeks. Tissue cultures were transported in thioglycollate broth and the samples were plated and incubated at 35°C both aerobic and anaerobic on 5% sheep blood, chocolate and MacConkey agar plates, and in thioglycollate broth for 14 days or until broth turned turbid. Subcultures were performed on the same primary plates. All microorganisms were routinely identified with matrix-assisted laser desorption ionization time of flight mass spectrometer MALDI-TOF (Bruker Daltronics, Bremen, Germany).

### DAIR

We performed a DAIR procedure in case of clinical suspicion of recurrence of infection in the acute postoperative period within 6 weeks of surgery or within 2 weeks of onset of an acute haematogenous infection of TKA. Up until 3 DAIR procedures were performed. Repetition of this treatment depended on the expected succes 13. The procedure was always perfomed by a complete arthrotomy and the polyethylene insert was always exchanged. Skin margins and sinus tracts were excised if present. Radical synovectomy and thorough lavage of the joint (using 3L of Betadine diluted Saline followed by 3L of Saline) were performed in each case.

### Outcome measures

To obtain the reinfection rate, we registered adverse events and the dates of all relevant surgeries, including reoperations (retention of implant) and re-revisions (exchange of implant) due to infection. We subdivided infections into new infections (new/other causative was cultured with respect to the index revision) and relapses (the same organism was cultured with respect to the index revision). To obtain the re-revision rate, we registered revisions due to any reason. We subdivided revisions into septic and aseptic revisions. We defined septic revision as removal of the prosthesis in a patient with a positive culture report from joint aspiration and/or preoperative or intraoperative histology consistent with infection and/or presence of a sinus tract. Reinfection and re-revision rates were assessed after 2 and 5 years and after mean follow-up time. To answer our third question we allocated patients into 3 groups: patients with negative cultures at reimplantation, patients with 1 positive culture and patients with 2 or more positive cultures with the same organism and compared their reinfection rates.

### Data analysis

A Fisher exact test was used to determine if reinfection and re-revision rates differed between patients with or without previous two-stage revision due to PJI. Reinfection rates were analysed after 2 and 5 years by calculating the cumulative incidence (and 95% confidence interval [CI]), accounting for the competing risk of death and patients lost to follow-up. A Chi-Square analysis was used to determine statistical differences between the groups with a different number of positive cultures at reimplantation. Significance level was set at α = .05. We determined Kaplan-Meier survivorships for reinfection, with diagnosis of a new infection or relapse as endpoint.

## Results

### Reinfection rate

**Figure 2:**
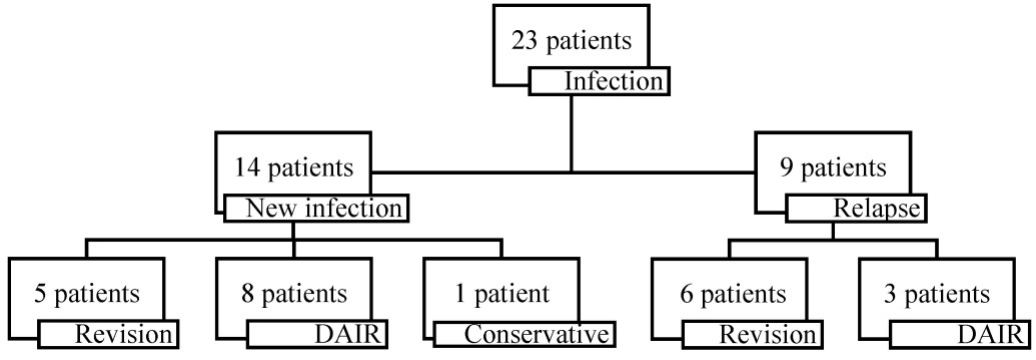
Reinfections, divided by new infections and relapses, and how reinfections were treated.

**Figure 3:**
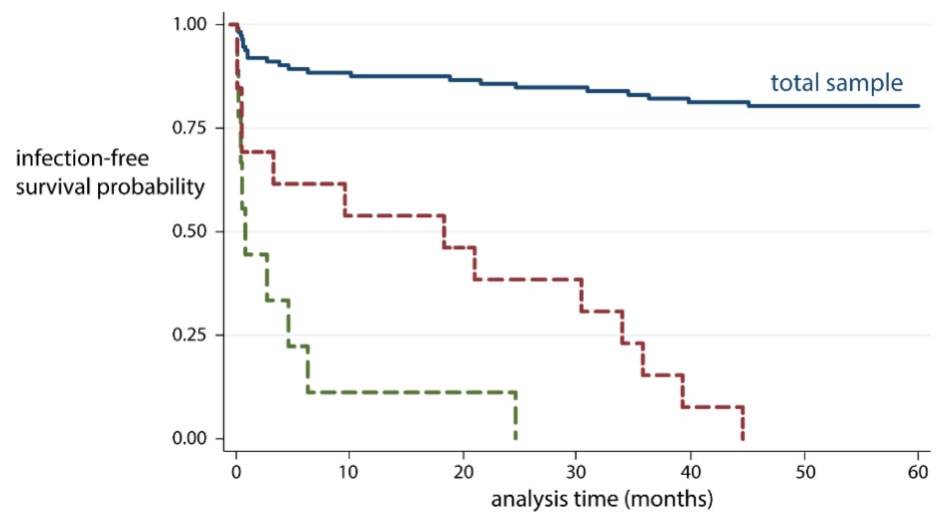
Survival with reinfection as an end point after two-stage revision of an infected TKA, stratified by no reinfection (blue), new infection (red) and relapse (green).

At the time of latest follow-up, 23 patients (23%) had experienced a reinfection (Figure 2). The mean time to reinfection after reimplantation was 12.2 months (SD 14.8) (Figure 3). Of the reinfections, 14/23 were new infections and 9/23 were relapses (Figure 2). Mean time to new infection was 17.4 months (SD 16.4) and 4.5 months (SD 7.4) to relapse. The cumulative incidence of reinfection was 14.3% (95%CI, 9.0% to 22.3%) at 2 years and 20.0% (95%CI, 13.7% to 28.8%) at 5 years. No recurrence of infection was seen after 5 years.

Of the new infections, 1 patient was treated conservatively with antibiotic treatment, after an acute haematogenous infection elsewhere. Eight patients could be treated with 1 or multiple DAIR procedures and 5 patients required re-revision surgery. Of the relapses, 3 patients could be treated with 1 or multiple DAIR procedures and 6 patients required re-revision surgery. Reinfection rates of patients who underwent a previous two-stage revision for PJI (4/20; 3 new infections and 1 relapse) were comparable to patients who did not underwent a previous two-stage revision (19/93) (p=1).

### Re-revision rate

After overall follow-up (range 5 to 14.3 years), 17 patients (17%) underwent re-revision surgery (6% at 2 years, and 9% at 5 years). Mean time to re-revision surgery was 33.7 months (SD 29.5). Eleven patients (11%) underwent re-revision surgery due to a new or recurrent infection. Mean time to septic re-revision was 22.5 months (SD 22.9).

Re-revision surgery for the new infections included arthrodesis (2), above-the-knee amputation (1), repeat two-stage revision (1) and repeat two-stage revision followed by above-the-knee amputation (1). Re-revision surgery for the relapses included repeat two-stage revision (3), arthrodesis (2) and above-the-knee amputation (1).

Six patients (6%) underwent re-revision surgery for aseptic reasons. Mean time to aseptic re-revision surgery was 56.0 months (SD 31.8). Re-revision surgery for aseptic reasons included 3 revisions of both components and 3 femoral revisions.

### Positive cultures at reimplantation

**Figure 4:**
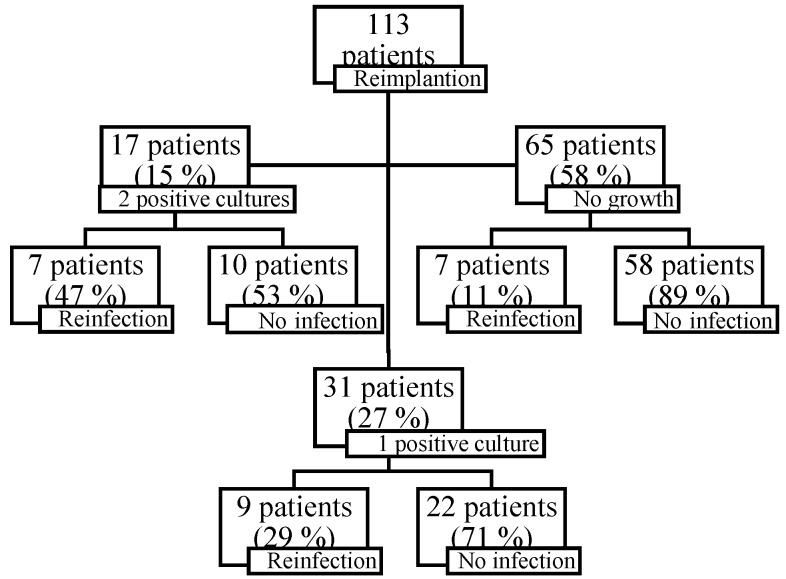
Reinfection rates divided by culture results at reimplantation.

Reinfection rates were different between these groups (χ2 9.67; p<0.01): 47% (7/47 with more than 2 positive cultures), 29% (9/31 with 1 positive culture) and 11% (7/65 with negative cultures) (Figure 4). Reinfection rates were higher in cases with positive cultures at reimplantation. Reinfection rate was higher in patients with 2 or more positive cultures compared to patients with less than 2 positive cultures (χ2 5.35; p=0.02). The reinfection rate in patients with 1 positive tissue culture was higher compared to patients without positive cultures (χ2 5.04; p=0.02). Two patients with an external fixator after first stage had a reinfection, both had 1 positive culture at reimplantation.

In the case of 2 or more positive cultures at reimplantation, a new microorganism was cultured in 13 cases and the same microorganism was cultured in 4 patients (see Table 3 for cultured organisms).

## Discussion

The purpose of this study was to examine the long-term results of two-stage revision for PJI following TKA. At long term follow-up 77% of the patients remained infection free. Implant survival was 83% at last follow-up moment. Reinfection rates were higher when positive cultures were observed at reimplantation.

Of our patients, 77% remained infection free, which is in line with most recent literature 9,14,15. On the other hand, reinfection rates up to 40% are also reported 7. We believe that a reinfection rate of 23% at long-term follow-up is acceptable, considering that treatment of an infected TKA with a two-stage revision protocol is challenged by an often extensive history of surgeries, underlying comorbidity, poor soft tissue quality, and organism virulence, biofilm formation and resistance profile.

Slightly more reinfections could be attributed to a new infection than to relapse of the treated infection. With the exception of 1 patient, relapse only occurred in the first 6 months following reimplantation. In contrast, infections with a newly cultivated organism were more evenly distributed over the first 5 years of follow-up. Acute hematogenous infections could be a possible explanation for this distribution of new infections. Multiple previous surgery, poor soft tissue and large metal implants are mentioned to increase the lifelong susceptibility for bacteremias to cause PJIs 16. No recurrence of infection was seen after 5 years. We do not expect the reinfection rate to rise with a longer follow-up, as our data and other studies have shown 9,10,14. Among other factors, previous revision surgery is a risk factor for reinfection 9. The current data set did not replicate that finding. In contrast to previous reports, we did not observe higher reinfection rates in patients with previous revision surgery for the same knee 9.

Implant survival in our data set is in line with comparable literature commenting on re-revision rates 9,10,17. Eleven patients (11%) received re-revision surgery for septic causes, the majority in the first four years following reimplantation. We do not expect this number to increase, since late recurrence of infection rarely occurs, as commented earlier. In case of an acute infection, our data substantiates that revision surgery for septic causes can successfully be prevented by (repeated) DAIR-procedures. In more than half of the patients with a reinfection, DAIR eradicated the infection and implant removal was not needed. DAIR has shown to be effective in acute postoperative (≤4 weeks postoperative) or acute haematogenous infections 13. Considering current guidelines, we believe a DAIR-procedure should be considered in patients without presence of a sinus tract who are within approximately 4 weeks of prosthesis implantation or less than 3 weeks of onset of infectious symptoms 6. Increased failure rates are associated with multiple DAIR-procedures 18, but alternative options require removal of implant. If surgical management is deemed impossible or the patient does not desire surgery, indefinite antibiotic suppressive therapy (AST) is the only remaining treatment option. In a recent study AST following PJI after total hip replacement was considered successful in 56.5% of the patients. High failure rates were seen in PJIs caused by S. aureus and in patients with an antibiotic-free period before the start of AST 19.

**Figure 5:**
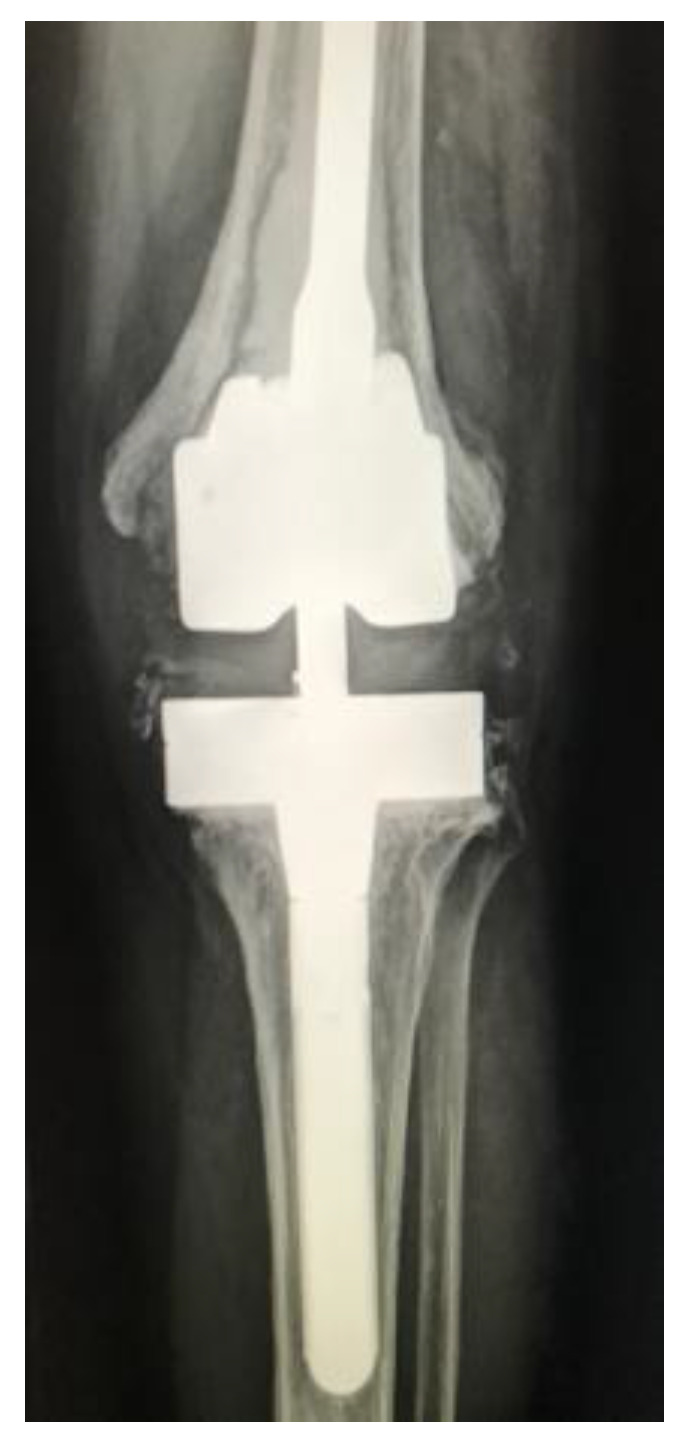
Aseptic loosening in not fully cemented Hinge Revision Total Knee Arthroplasty.

Our re-revision rate for aseptic causes (6%) is lower than other recent reports on two-stage revisions with long-term follow-up 9,10. Analysing our patients with aseptic loosening it seems that radiographically (Figure 5) we have learned that using a Hinge Revision TKA without fully cemented stems and not providing primary and secondary stability in two out of three zones (zonal fixation as described by Morgan-Jones et al.) contributed to the cause of aseptic loosening after reimplantation 20.

The results of this study are consistent with a recent meta-analysis that reveals that at least 1 positive culture at reimplantation increases the risk of reinfection 21. In contrast to our study, this meta-analysis has not analysed the failure rates on a long-term (restricted to 4 years). We did not observe recurrence of infection after 5 years of follow-up. If reimplantation cultures were positive, a new microorganism was cultured in the majority of the patients in our cohort. Reinfection rates in our study are higher with 1 positive culture result, compared to culture negative reimplantation. This is in line with previous reports 22,23. Recently, a multicenter randomized controlled trial suggested that addition of 3-month oral antibiotics following reimplantation improved the infection-free survival 24.

We recognized the limitations to our study. While we collected the data prospectively, the study was a retrospective analysis and had the implicated limitations. This includes inability to obtain all data that could be helpful. We noticed the high percentage of culture negative patients and thus, since we were unable to collect all data on minor criteria, only 58% of the patients were diagnosed with infection based on the major MSIS-criteria. In the study by Petis et al. 33% of the patients were diagnosed with infection by minor criteria only 9. Oral antibiotic therapy has not always been registered in our medical database, therefore we were unable to collect all data on patients that were treated with suppressive antibiotics following two-stage revision and on patients with recurrence of infection treated with antibiotics alone. Therefore we possibly underestimated the reinfection rate. In a recent study the percentage of infections treated with suppressive antibiotics was 2% 9. Secondly, we did not perform a correction analysis on a number of confounding factors, such as the use of antibiotic spacers and external fixators, operative time, smoking status and patient comorbidities or BMI. Previous reports have identified that these factors were predictive for reinfection 9,14,25. The wide variety of bacteria identified also made it impossible to carry out statistical analysis on different microorganisms. Therefore we were unable to analyze if specific pathogens, such as *Enterococcus species*, were more difficult to eradicate 14. The antibiotic treatment protocol has changed in our institution. Cessation of antibiotic treatment prior to reimplantation is not performed. Currently, antibiotic treatment is continued until reimplantation.

In conclusion, treatment of a (suspected) infection of a TKA by a two-stage revision had acceptable results based on re-revision and re-infection rates in the long term (>5 years), resembling the short-term results (<2 years). Focussing on the cultures at the index two-stage revision, episodes of new and recurrent infections during follow-up were almost equally divided. Reinfection rates were higher in cases with positive cultures at reimplantation. Patients should be counselled appropriately in this particular situation.

## Figures and Tables

**Table 1:**
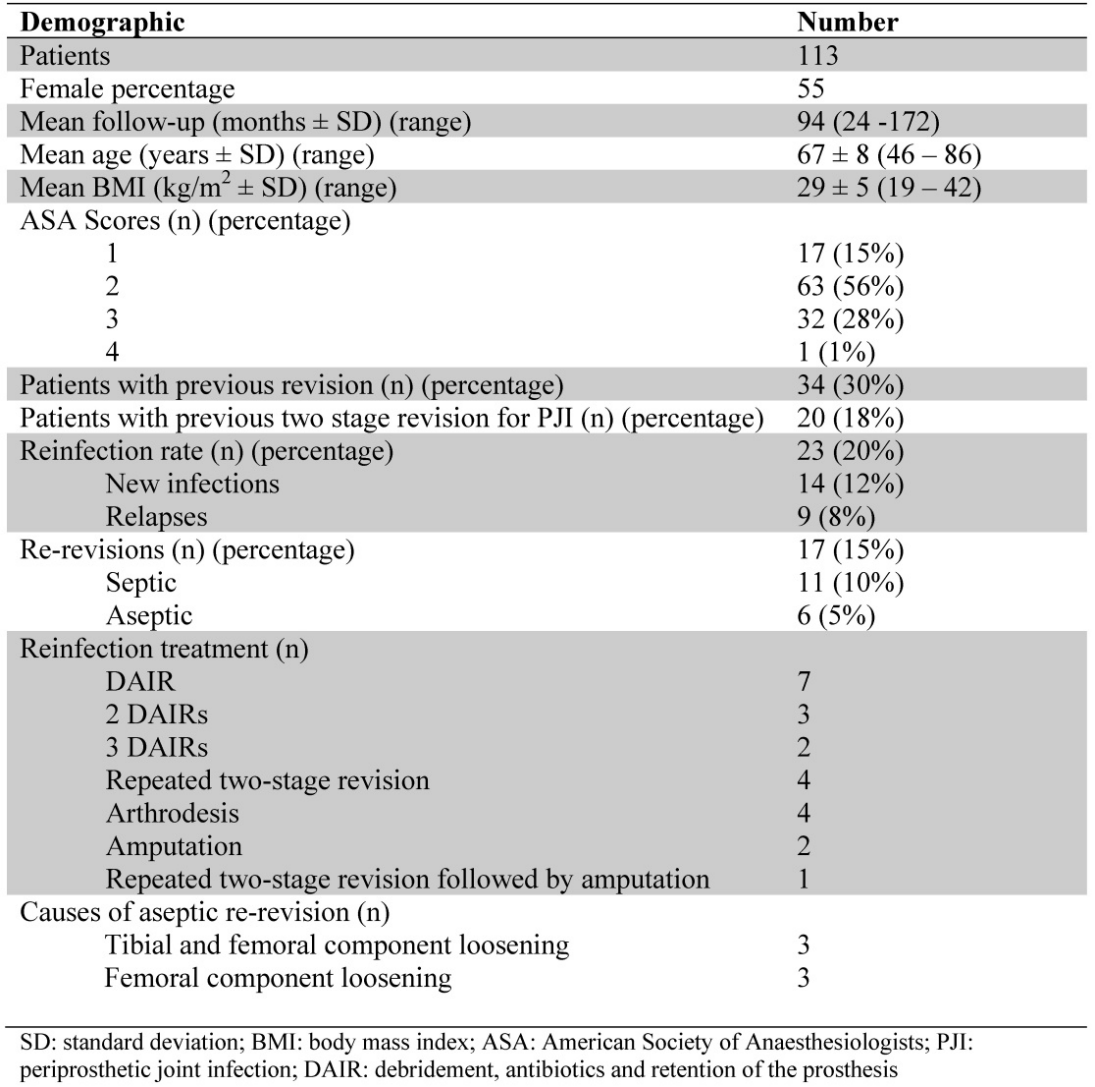
Patient demographics, reinfection rates, re-revision rates, reinfection treatment and causes for aseptic re-revision

**Table 2:**
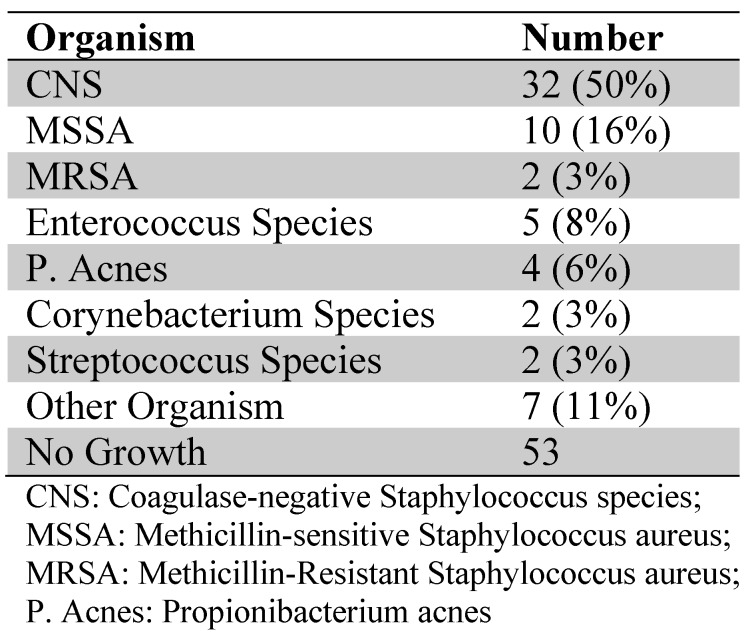
Organisms cultured preoperatively or at implant removal

**Table 3:**
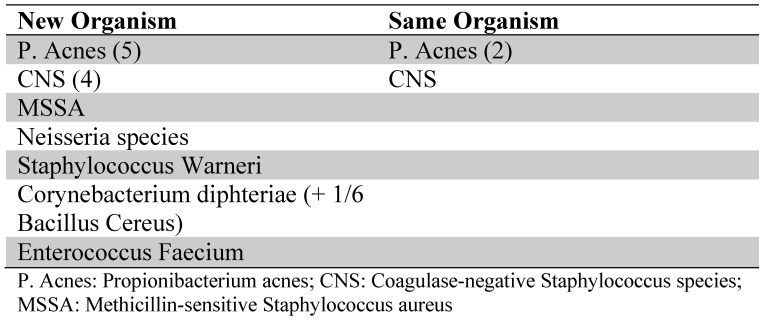
Organisms cultured at reimplantion with respect to the index operation
